# Strategies to reduce the onset of sleeve gastrectomy associated bone loss (STRONG BONES): Trial design and methods

**DOI:** 10.1016/j.conctc.2023.101181

**Published:** 2023-07-04

**Authors:** Joshua R. Stapleton, Jamy D. Ard, Daniel P. Beavers, Lori S. Cogdill, Adolfo Z. Fernandez, Marjorie J. Howard, Jamie N. Justice, S. Delanie Lynch, Jovita J. Newman, Ashley A. Weaver, Kristen M. Beavers

**Affiliations:** a Department of Biomedical Engineering, Wake Forest University School of Medicine, Winston-Salem, NC, USA; b Weight Management Center, Atrium Health Wake Forest Baptist, Winston-Salem, NC, USA; c Department of Statistical Sciences, Wake Forest University, Winston-Salem, NC, USA; d Department of Health and Exercise Science, Wake Forest University, Winston-Salem, NC, USA; e Department of Biostatistics and Data Science, Wake Forest University School of Medicine, Winston-Salem, NC, USA; f Department of Internal Medicine, Section on Gerontology and Geriatric Medicine, Wake Forest University School of Medicine, Winston-Salem, NC, USA; g Department of Epidemiology and Prevention, Wake Forest University School of Medicine, Winston-Salem, NC, USA

**Keywords:** Bone mineral density, Bariatric surgery, Older adults, Weight loss, Bisphosphonate

## Abstract

**Background:**

Despite recognized improvements in obesity-related comorbidities, mounting evidence implicates surgical weight loss in the onset of skeletal fragility. Sleeve gastrectomy (SG) is the most commonly performed bariatric procedure and is associated with 3–7% axial bone loss in the year following surgery. Bisphosphonates are FDA-approved medications for the prevention and treatment of age-related bone loss and may represent a strategy to reduce bone loss following SG surgery.

**Methods:**

The Strategies to Reduce the Onset of Sleeve Gastrectomy Associated Bone Loss (STRONG BONES) trial (NCT04922333) is designed to definitively test whether monthly administration of the bisphosphonate, risedronate, for six months can effectively counter SG-associated bone loss. Approximately 120 middle-aged and older (≥40 years) SG patients will be randomized to six months of risedronate or placebo treatment, with skeletal outcomes assessed at baseline, six, and 12-months post-surgery. The primary outcome of the trial is 12-month change in total hip areal bone mineral density (aBMD), measured by dual energy x-ray absorptiometry (DXA). This will be complemented by DXA-acquired aBMD assessment at other skeletal sites and quantitative computed tomography (QCT) derived changes in bone quality. Change in muscle mass and function will also be assessed, as well as biomarkers of bone health, turnover, and crosstalk, providing mechanistic insight into intervention-related changes to the bone-muscle unit.

**Discussion:**

Results from the STRONG BONES trial have the potential to influence current clinical practice by determining the ability of bisphosphonate use to mitigate bone loss and concomitant fracture risk in middle-aged and older SG patients.

## 1 Introduction

Over the past decade, severe obesity [body mass index (BMI) ≥40 kg/m^2^] prevalence has steadily increased in the United States, with recent estimates showing 11% of middle-aged (40–59 years) and 6% of older (60+ years) adults affected. [1] Bariatric surgery is increasingly utilized to treat severe obesity, with the sleeve gastrectomy (SG) procedure accounting for ∼60% of all bariatric procedures. [2] Although SG is effective at reducing weight and comorbidities associated with obesity, evidence suggests a 3–7% concomitant reduction in areal bone mineral density (aBMD). [3] Reviews of fracture risk secondary to bariatric procedures indicate a higher likelihood of fracture. [[4], [5], [6]] In recognition of this clinical conundrum, the American Society for Metabolic and Bariatric Surgery (ASMBS) issued a position statement in 2020 calling for additional randomized data to better determine optimal interventions and treatments aimed at minimizing fracture risk in bariatric surgery patients. [7]

**Fig. 1:**
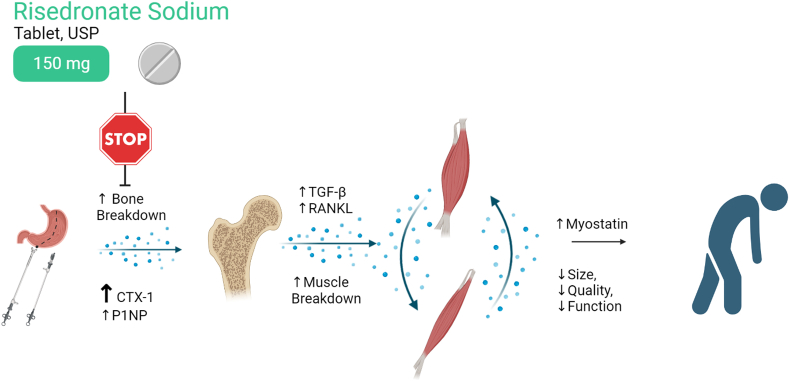
Hypothesized mechanism of lean tissue sparing effect from risedronate.

Once-monthly risedronate [8] is an oral bisphosphonate prescribed to prevent and treat bone loss [[8], [9], [10]] by decreasing osteoclast activity, thereby slowing bone resorption. [11,12] As bone loss following SG is driven by increases in resorption (versus declines in formation), [13] it is reasonable to hypothesize that SG-associated bone loss [14] could be countered by osteoclast inhibition with risedronate. Indeed, pilot data support this premise, demonstrating that 6-months of risedronate treatment is feasible and likely effective in reducing SG-associated bone loss and resorption, as compared to placebo. [15,16] Data from this pilot trial also signal a lean mass sparing effect with risedronate use. This novel finding aligns with data from murine models of clinical pathology [[17], [18], [19]] and limited observational data in humans, [20,21] which we hypothesize may be due to a blunting of SG-associated osteokine release (Fig. 1). The pilot trial makes use of short-term therapy during the most active weight loss phase to minimize patient exposure and the cost of therapy. An appropriately powered trial will support updates to postoperative care for SG patients, [22] while also providing a unique platform to investigate mechanisms of bone-muscle crosstalk. [7,22]

The main objective of the Strategies to Reduce the Onset of Sleeve Gastrectomy Associated Bone Loss (STRONG BONES) trial is to definitively test whether risedronate use can effectively counter SG-associated musculoskeletal tissue loss. To address this clinical question, approximately 120 middle aged and older (≥40 years) SG patients will be randomized to a six-month treatment of risedronate or placebo with outcomes assessments taken baseline, six, and 12-months. The primary outcome is total change in hip aBMD (measured by dual energy x-ray absorptiometry [DXA]) due to its robust change following surgery [3] and clinical utility in fracture risk assessment. [23] We hypothesize that patients assigned to risedronate will better preserve total hip aBMD than patients assigned to placebo. Secondarily, the trial will explore treatment-related change in DXA-acquired aBMD assessment at other skeletal sites; quantitative computed tomography (QCT) derived changes in bone quality; muscle mass and function; and biomarkers of bone health, turnover, and crosstalk.

## 2 Methods

### 2.1 Overview

**Fig. 2:**
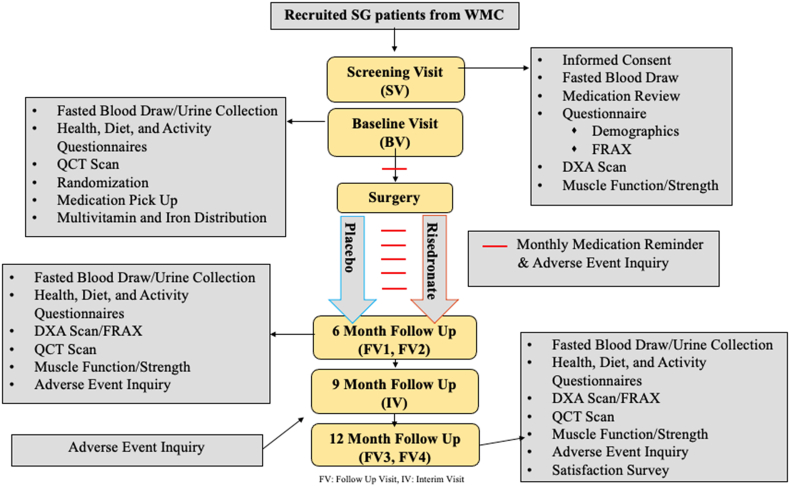
Outline of study flow. FRAX: Fracture Risk Assessment Tool; DXA: Dual-Energy X-Ray Absorptiometry; QCT: Quantitative Computed Tomography; FV: Follow Up Visit; IV: Interim Visit.

The STRONG BONES trial has been approved by the Wake Forest University Institutional Review Board (IRB00074763) and is registered on clinicaltrials.gov (NCT04922333) as a single site, interventional, placebo-controlled, double-blind clinical trial. An overview of the study design is provided in Fig. 2. Health Insurance Portability and Accountability Act (HIPAA) authorization and informed written consent will be obtained from all patients prior to enrollment and data collection.

### 2.2 Patient population, recruitment, screening, and randomization

Potential patients will be recruited from the Atrium Health Wake Forest Baptist Weight Management Center prior to their surgery date and in accordance with the eligibility criteria outlined in Table 1, which serves to verify our target population and eliminate those who could be adversely affected by the intervention. Women of child-bearing potential are counseled about willingness to use effective contraceptives to prevent pregnancy during the 12-month observation period**.** After completing all screening assessments, eligible patients will be randomized to either risedronate or placebo treatment via computer-generated permuted block randomization scheme with blocks of random size (2, 4, 6, or 8) and stratified by sex.

**Table 1:** Inclusion and exclusion criteria.

Criteria	Inclusion Criteria	Exclusion Criteria
Written Consent	Sign and agree	Do not sign/agree
Age (years)	≥40	<40
Obesity Stats	BMI ≥40 kg/m^2^ or BMI ≥35 kg/m^2^	Weight >450 lbs [204 kg] (DXA/CT limit)
	with ≥1 obesity related complication including:	
	Hypertension (>160/90 mm Hg);	
	Type II diabetes; Hyperlipidemia; Obstructive Sleep Apnea	
Allergy		Known allergy to bisphosphonates
Surgical Status	Cleared by attending physician for SG procedure	
Transportation	Able and willing to provide own transportation to screening and assessment visits	
Blood Markers	Serum calcium (8.6–10.2 mg/dL)	Serum calcium outside of normal range
	PTH (15–65 pg/mL)	PTH outside of normal range
	eGFR ≥45 mL/min/1.73 m [2]	eGFR <45 mL/min/1.73 m [2]
	25-hydroxyvitamin D levels ≥20 ng/mL	25-hydroxyvitamin D levels <20 ng/mL
Mobility		Dependent on cane/walker
Medication use		Current or prior (within the past year) use of growth hormones; oral steroids for more than 6 consecutive days in the past 6 months or any use of prescription osteoporosis medications
		Unstable gastric reflux requiring two or more additional doses per month of anti-reflux medication
Research participation		Participation in another conflicting research study
		Unable to position on DXA or CT scanner independently
Medical Contraindications		Existing osteoporosis; Planned hip surgery or existing bilateral hip implants; Pregnancy or potential pregnancy (positive pregnancy test or unwilling to use a highly effective contraception method during active study phase); Esophageal abnormalities; Increased risk of ulceration; Paget's disease; Primary hyperparathyroidism; Hyper/hypothyroidism; Severe liver disease; History of malignancy within 5 years; Use of bone-active medications; Sensitivity to bisphosphonates; Extensive dental work involving extraction or dental implant within the past 2 months or planned upcoming 6 months; Existing risk of osteonecrosis of the jaw or atypical femur fracture; Deemed unfit for any reason by study physician/principal investigator

BMI: Body Mass Index; kg: Kilogram; lb: Pound; DXA: Dual-Energy X-Ray Absorptiometry; CT: Computed Tomography; mm HG: millimeters mercury; SG: Sleeve Gastrectomy; mg: milligram; dL: Deciliter; PTH: Parathyroid Hormone; pg: picogram; eGFR: Estimated Glomerular Filtration Rate; min: minute; m: meters; ng: nanogram.

### 2.3 Surgery and interventions

#### 2.3.1 Surgery requirements

All patients will adhere to this clinic visit schedule post-surgery: overnight hospital stay; 30-day nutrition and surgeon follow-up; three-month nutrition and blood draw follow-up; six-month surgeon, resting metabolic rate, and exercise follow-up; nine-month nutrition follow-up; and 12-month surgeon and resting metabolic rate follow-up. ASMBS recommendations for perioperative nutrition, metabolic, and nonsurgical support of bariatric surgery patients will be followed. [22] All patients will be provided a six-month supply of daily Celebrate Essential Multi 2-in-1 chewable tablet (providing ∼30% daily calcium and ∼95% daily vitamin D) and sex-dependent iron supplement to ensure micronutrient needs are met throughout the active study period.

#### 2.3.2 Intervention description

Over-encapsulated tablets containing 150 mg risedronate or placebo will be dispensed from the Wake Forest University School of Medicine Investigational Drug Service to each participant post randomization at their baseline assessment visit. Patients will receive instructions to take the first dose 3–7 days prior to their procedure and will be instructed to follow label instructions (i.e., take medication with 6–8 oz of plain water; remain upright for the next 30 min; avoid taking vitamins, mineral supplements, or antacids at the same time). Monthly text or phone reminders for medication use, potential pregnancy (if applicable) and any new adverse events will be performed by the study team. A formal pill count will occur at six months based on the returned pill bottle. In the event of a missed dose (e.g., the participant was unable to take the medication on the planned date), the participant will be instructed to take the medication as near to the planned date as possible. However, if the next month's scheduled dose is within seven days, the participant will be instructed to wait until the next month's scheduled dose. In either scenario, the participant will resume the originally scheduled day of the month on the once-monthly administration schedule. Deviations from the planned date will be noted and missed doses will be corroborated with pill count assessments.

#### 2.3.3 Assessments

All assessments will be conducted by trained and blinded assessors, with in-person visits occurring at baseline, six, and 12 months (see Table 2 timeline). Briefly, demographic data will be assessed at the screening visit. Questionnaires, physical function testing, safety assessment and biomarker specimens, and medical imaging will be obtained at the baseline, six, and 12-month visits.

**Table 2:** Assessment chart indicating data collection and sampling for each visit.

Week:	−6 weeks to −3 days		−1 to +23	25		38	52	
Visit Window:			−3 to −7 days	±15 days		±15 days	±15 days	
	SV	BV	INT	FV1	FV2	IV	FV3	FV4
Informed Consent	x							
Demographic Characteristics	x							
Medical History/Medication Use	x		x	x			x	
Vitals: Height/Weight/Blood Pressure	x			x			x	
BMD/FRAX Questionnaire	x			x			x	
SF-36 Questionnaire		x			x			x
DXA Assessment (regional BMD, total body composition)	x			x			x	
QCT Assessment (hip/spine bone and muscle quantity and quality)		x			x			x
Fasting Blood Draw for real-time safety (vitamin D, PTH, CMP, A1C, FSH) and storage (biomarkers of bone turnover and muscle bone crosstalk, and gut hormones)	x	x			x			x
Fasted Urine Collection		x			x			x
Pregnancy Testing (all pre-menopausal women who have not had a hysterectomy)	x	x		x	x		x	x
Muscle Function/Strength (400-m walk, stair climb, knee extension strength)	x			x			x	
Randomization, Drug, and Multivitamin Dispensation		x						
Monthly Medication Compliance Reminders			x					
Unused Drug Return, Pill Count for Compliance Assessment					x			
Vioscreen FFQ		x			x			x
CHAMPS Physical Activity Questionnaire		x			x			x
Adverse Events			x	x	x	x	x	x
Exit Survey/Participant Satisfaction								x

BMD: Bone Mineral Density; QCT: Quantitative Computed Tomography; PTH: Parathyroid Hormone; CMP: Complete Metabolic Panel; CHAMPS: Community Health Activities Model Program for Seniors; WFU: Wake Forest University; WFUSM: Wake Forest University School of Medicine; SV: Screening Visit; BV: Baseline Visit; INT: Intervention; IV: Interim Visit; FV: Follow Up Visit.

### 2.4 Primary outcome: DXA-derived total hip areal bone mineral density

This study is powered to detect significant group differences in change in total hip aBMD assessed by DXA over 12 months. The 12-month duration was selected to ensure adequate bone remodeling time in response to the intervention, [24] with inclusion of the six-month assessment to increase study power and allow for a midpoint safety assessment on all patients. DXA scans of the hip (as well as the lumbar spine, radius, and whole-body for secondary outcomes; see Section 2.4) will be acquired on an Hologic Horizon A device (Bedford, MA) and read by an International Society for Clinical Densitometry trained DXA technologist. Scans will be examined for proper patient positioning and exclusion of artifacts from the measured region; re-scanning will be performed as necessary. Daily quality control scans will be obtained with a calibration phantom, with repeat phantom scans if results are >2 standard deviations from baseline.

### 2.5 Secondary outcomes: DXA, QCT, and muscle function/strength assessments

To increase clinical utility and mechanistic understanding, we will assess intervention effectiveness on DXA-acquired metrics collected at different skeletal sites (aBMD of the femoral neck, lumbar spine and distal radius), as well as appendicular lean mass, total body fat, and visceral fat, using a mirroring protocol if necessary. [25] Secondary outcomes will also include QCT-acquired metrics of bone and muscle quantity and quality, as well as muscle function and strength assessments that are sensitive to intensive weight loss and predictive of fall risk.

#### 2.5.1 Quantitative computed tomography (QCT) derived measures

Helical QCT scans of the L1-L5 vertebrae and femurs (proximal to mid-shaft) will be acquired at baseline, six, and 12 months on a GE 64-slice PET/CT Discovery MI scanner at 120 kV, 50-cm field of view, automatic exposure (target noise index 20 HU), 0.625-mm slice thickness, 1:1 pitch, and an abdomen reconstruction filter with secondary reconstruction to 2.5-mm (abdomen filter) and 0.625-mm (bone filter). The Mindways Model 3 CT calibration phantom and bolus bag (Mindways Software, Inc., Austin, TX) will be positioned under each participant and imaged in every scan to calibrate volumetric BMD (vBMD). Quality assurance scans will be performed monthly to monitor operational characteristics of the scanner and the phantom.

##### 2.5.1.1 Volumetric BMD (vBMD)

Lumbar vertebra trabecular vBMD and proximal femur (femoral neck, trochanter, intertrochanter, and total hip) trabecular, cortical, and integral vBMD will be measured in QCT scans using QCT Pro™ software (Mindways Inc., Austin, TX). Mean vertebral, femoral neck, trochanter, intertrochanter, and total hip vBMD will be calibrated in terms of equivalent aqueous K_2_HPO [4] density values in the 5-port phantom. [26]

##### 2.5.1.2 Cortical thickness

The proximal femur will be manually segmented from CT scans. Cortical thickness will be measured from the segmentations using a cortical density-based algorithm implemented in Stradview (University of Cambridge, UK). [[27], [28], [29]] Each participant's baseline, six, and 12-month cortical thickness maps will be rigidly registered using the iterative closest point algorithm to measure global and localized cortical thickness changes. [30]

##### 2.5.1.3 Finite-element (FE) modeling-derived bone sstrength

**Fig. 3:**
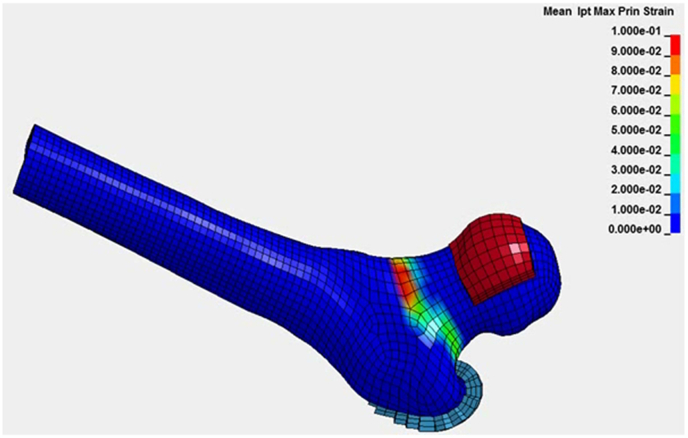
Finite element hip bone strength (high femoral neck strains in red). (For interpretation of the references to colour in this figure legend, the reader is referred to the Web version of this article.)

Subject-specific FE models of the proximal femur at baseline, six, and 12 months will be developed. [31,32] Subject-derived material properties will be implemented using vBMD for elasticity and variable cortical thickness to improve accuracy. [33,34] Bone strength and fracture risk will be estimated with simulated tests of a sideways fall (Fig. 3). [35] Simulations will be performed using the LS-Dyna implicit FE solver (LSTC, Livermore, CA) [32] where bone strength will be defined as the peak force between the impactor and the femoral head. Strain-based criteria have proved effective to predict bone fracture. [36]

#### 2.5.2 Muscle area and quality

Mid-thigh muscle cross-sectional area (CSA), muscle density (measured by Hounsfield Unit [HU] attenuation), and intermuscular fat area of the mid-thigh and total trunk muscles at the L3 vertebra level will also be assessed. The mid-thigh position will be defined as the midpoint between the superior aspect of the greater trochanter and the inferior aspect of the lateral condyle measured on an anterior-posterior scout of the entire femur. All trunk muscles will be defined from a CT slice at the midpoint of the L3 vertebral body. Muscle and intermuscular fat areas will be segmented from CT by thresholding for fat (−190 to −30 HU) and muscle (−29 to 150 HU) and then manually refining the segmentation as needed. Abdominal visceral and subcutaneous fat area at the mid-L3 vertebra level will be measured and segmented from CT by thresholding (−190 to −30 HU) and then manually refining the segmentations.

#### 2.5.3 Muscle function/strength

The fast 400-m walk test will be used to measure gait speed. [37] In addition, stair climbing ability will be assessed by the fastest time to climb a 12-step staircase (height 213.36 cm) in two trials. Knee extension strength (peak torque in Newton meters [Nm]) will be measured on the same side as the DXA scan (non-dominant unless there is hardware, fracture or non-weight-bearing >6 weeks in the last 12 months) via isokinetic dynamometry (Humac Norm, CSMi, Massachusetts). These tests are proven to be sensitive to intensive weight loss [38,39] and predictive of fall and future fracture risk. [40,41]

### 2.6 Tertiary outcomes: biomarkers

#### 2.6.1 Biomarkers of bone health, bone turnover, bone-muscle crosstalk and gut hormones

To elucidate mechanism contributing to change in imaging parameters of bone and muscle, we propose to investigate the impact of SG alone and with risedronate use on select biomarkers of bone health, [42,43] bone turnover, [44] and bone-muscle crosstalk. [[45], [46], [47]] Additionally, we include biomarkers of select circulating gut hormones to explore the potential role of SG-associated gut hormone change on secondary bone resorption/loss. [[48], [49], [50]] Serum, plasma, whole blood in DNA/RNA stabilizing tubes, and urine will be collected and stored for use in future ancillary studies. After processing, aliquots will be stored at −70 °C or colder until analysis. Total blood volume collected over the course of the study is estimated to be 193.5 ml.

### 2.7 Covariate assessment

Demographic data will be ascertained based on participant self-report at baseline. Medical information on prior and existing co-morbidities, falls, and hospitalizations will be ascertained by self-report and querying the medical record at all visits. Similarly, the Fracture Risk Assessment Tool [FRAX (v 4.1)] will be used to assess 10-year major osteoporotic and hip fracture risk on all patients, [51] the Short Form Health Survey (SF-36), [52] the Vioscreen Dietary Assessment Tool, [53] and the Community Health Activities Model Programs for Seniors (CHAMPS) [54] will be administered as self-reported measures of health.

Protocol adherence will be measured using monthly self-reported pill counts, and a formal six-month pill count. A monthly health status questionnaire will facilitate standardized collection of AEs and concomitant medication usage, in accordance with our data and safety monitoring plan.

### 2.8 Data and statistics

#### 2.8.1 Data management and statistical approach

In accordance with best practices, data will be retained in a secure, password-protected electronic database. Dynamic reports and statistical analyses will monitor data quality. A participant-based inventory system will track recruitment, retention, adherence, and missing data from entry through exit, close-out, and final lock-down. Primary analyses will use intention-to-treat principles per CONSORT guidelines, with secondary analysis following a per protocol approach (including patients who are >80% compliant with the medication protocol) to assess whether protocol compliance affects observed intent-to-treat (ITT) results. Serious AEs will be reported within 48 h to maintain up to-date safety information for reporting to the Data and Safety Monitoring Board. All data will undergo range checks at the time of data entry and will be examined monthly by histograms and bivariate scatterplots to check for inconsistencies, unusual data needing further verification, and outliers. Regression diagnostics and exploratory analyses will be performed to find appropriate transformations of variables if needed. Order of priority in choosing a transformation will be to satisfy: 1) linearity, 2) homogeneity, and 3) normality assumptions. We will attempt to identify baseline covariates that predict attrition and compliance; and if identified, secondary analyses may need to incorporate stratification by these factors to decrease bias. As recommended by the National Academy of Sciences, [55] if the outcome is missing at random, we will attempt to identify baseline covariates that predict attrition and use these covariates to impute missing data based on multiple imputation. Sensitivity analyses, using pattern mixture models, will explore the effect of missing outcomes on inference if not at random.

#### 2.8.2 Sample size and power

Our preliminary data suggests that, following SG, total hip aBMD declines by approximately 6.7% (−0.072 g/cm^2^) in 12 months from a baseline (SD) value of 1.06 (0.13) g/cm^2^. [16] We conservatively hypothesize that risedronate use will attenuate total hip aBMD change by ∼43% [−0.03 g/cm^2^, i.e., risedronate treatment difference (improvement) of 0.023 g/cm^2^ or 2.2% of baseline, which is considered a clinically meaningful (i.e., 1.4–3.2%) amount [23]]. With 120 patients, we will have 92% power to detect a 0.023 g/cm^2^ difference between groups, based on a two-tailed *t*-test at α = 0.05 using common group standard deviations of 0.033 g/cm^2^ assuming 80% retention (87.5% observed at six months in the pilot RCT [15]), or 48 evaluable observations per group (Table 3).

**Table 3:** Detectable 12-month differences for 80% power for the primary outcome and key secondary outcomes.

Outcome	Baseline Mean	SD of change	Detectable mean difference (% of BL)	Power
Total Hip aBMD (g/cm^2^)	1.06	0.033	0.023 (2.2%)	92%
Femoral Neck aBMD (g/cm^2^)	0.903	0.040	0.023 (2.6%)	80%
Appendicular Lean Mass (kg)	23.6	1.57	0.907 (3.8%)	80%
Trabecular Hip vBMD (g/cm^3^)	0.283	0.02	0.012 (4.1%)	80%
Femoral Bone Strength (kN)	2.044	0.065	0.037 (1.8%)	80%
Fast-paced gait speed (m/s)	1.20	0.15	0.087 (7.2%)	80%

BL: Baseline. *Assumes comparisons at α = 0.05 and 80% retention at 12 months (n = 48/group evaluable).

Analyses at secondary skeletal sites include measurements procured using DXA and QCT and select physical performance measures. Based on the sample size used to justify the primary aims, we present the detectable mean differences for key secondary outcomes using 80% power assuming 80% retention at 12 months and each comparison using a two-tailed *t*-test at α = 0.05 (Table 3). Although DXA-acquired aBMD has the most clinical relevance, these secondary outcomes require modest changes to achieve statistical significance at 80% power and, more importantly, can help identify regions of interest or additional variables for future trials, as well as insights for understanding potential mechanisms for the consequences of SG with or without risedronate treatment on musculoskeletal health outcomes.

Tertiary aims will explore mechanisms underlying SG-associated bone loss with and without risedronate use; thus, it is not formally powered due to the exploratory nature of the analyses. However, a sample of 120 per combined treatment group (48/group evaluable, considering attrition) has 80% power to detect a medium biomarker effect size of 0.578, using a two-tailed test at α = 0.05. Exploratory comparisons will be based primarily on nominal p-values and will apply false discovery rate adjustments [56] to account for multiple outcomes.

#### 2.8.3 Statistical analysis plan

The primary aim for comparisons of total hip aBMD at 12 months will be tested using a linear mixed model fit with total hip aBMD as the primary outcome and the following independent variables: visit (six and 12-months), treatment effect indicator (1 for risedronate; 0 for placebo), the treatment by visit interaction, participant sex (to account for randomization strata and sex as a relevant biologic variable), and baseline total hip aBMD value, assuming an unstructured covariance. The effect of the intervention at 12 months will be estimated using a contrast statement to test the 12-month treatment group difference using a 0.05 level of significance. Additional analyses will test the short-term effect by estimating the six-month treatment effect from the same model. Per protocol sensitivity analyses will be conducted similar to primary analyses, except using only the subset of patients who took at least five of the six prescribed pills (risedronate or placebo). The secondary aim outcome variables will be tested similarly as the first, separately modeling each outcome using six and 12-month outcomes as the dependent variables of the mixed linear model and all other covariates the same, except respective baseline values will be used in place of baseline total hip aBMD. Treatment effects at 12 months, the comparisons of primary interest, will be tested at a two-tailed α = 0.05 level using contrast statements. All models will use completers only, noting that mixed models with baseline covariate adjustments are fairly robust to missing data; [57,58] however, sensitivity analyses will be conducted to ensure results are not biased due to differential missing data. Tertiary aim treatment effects will use the same models as the first two aims changing only the outcome and baseline covariate and treatment effects will be tested at the α = 0.05 level. Additional analyses will explore whether changes in biomarkers mediate the relationship between risedronate and BMD. Our pilot data do not suggest that we will see clinically meaningful differences in treatment effects by race/ethnicity or sex. However, to explore the possibility of differential treatment effects, we will include sensitivity analyses using race/ethnicity or sex as an interaction variable with treatment assignment to determine whether treatment effects differ across race/ethnicity or sex. All analyses will be conducted using SAS v9.4 (Cary, NC) and R software.

## 3 Discussion

The STRONG BONES trial is designed to definitively test whether musculoskeletal tissue loss following SG surgery can be mitigated through transient bisphosphonate therapy. Increasing utilization of SG to treat severe obesity, alongside increasing awareness of the skeletal complications of bariatric surgery, underscores the timeliness of this research question. If confirmed, data from this trial will present risedronate as a potential surgical adjuvant, aimed at maximizing the cardiometabolic benefits of SG surgery, while minimizing potential harm to the musculoskeletal system.

Bisphosphonates are a class of drugs that have been used commonly for more than two decades for the treatment and prevention of osteoporosis. As a class, these medications are particularly well absorbed in the trabecular region of the femoral neck and spine, [12] and have a potent effect on bone remodeling by inhibiting the breakdown of hydroxyapatite (the primary structural component of bone). [59] We specifically selected risedronate due to its efficacy, safety, [60,61] once-monthly dosing regimen, and low cost. [62] Together, the few reported AEs (5 total; 3.7% AE rate), high degree of protocol adherence (100% adherence among completers; 92% when non-completers were included), [15] and signal for total hip aBMD sparing [risedronate: -0.028 g/cm^2^ (−0.049, −0.006) vs. placebo: -0.047 g/cm^2^ (−0.063, −0.030)] at six months in the pilot study [15,16] provided impetus for the STRONG BONES trial primary aim and hypothesis.

That said, bariatric surgery patients are at heightened risk of developing hypocalcemia, [63] which may be elevated with concomitant bisphosphonate use. [64] Fortunately, hypocalcemia was not observed during the pilot study, however we still have planned safety measures to mitigate risk of this serious complication. All participants are screened at baseline to ensure serum calcium is within the normal range (8.6–10.2 mg/dL) and vitamin D levels are non-deficient (>20 ng/mL). Once enrolled, and in addition to nutrition counseling from their surgical team, all study participants receive a 6-month supply of the Celebrate Essential Multi 2-in-1 multivitamin, which provides ∼30% and ∼95% of the RDI for calcium and vitamin D, respectively (see *Section 2.2.1*). Additionally, routine post-operative clinical visits are scheduled at 1- and 3-months post procedure, where serologic markers are assessed; and, a fasting blood draw will be collected at the 6- and 12-month study visit to assess safety-related labs (including: calcium, vitamin D, eGFR, and parathyroid hormone). Finally, as a NIAMS-funded clinical trial, this study is overseen by an external, 5-member data safety monitoring board, which meets biannually to help the study team ensure participant safety and data integrity are prioritized throughout the trial.

This trial is also designed to offer additional insight into the biology of SG-associated lean mass loss, as well as potential counteractant effects of risedronate use. We specifically build off the appendicular lean mass sparing signal observed in the pilot study [risedronate: -1.2 kg (−2.3, −0.1) vs placebo: -2.1 kg (−3.0, −1.2)] to explore whether the blunting of SG-associated bone resorption can preserve muscle via bone-muscle crosstalk. As the field of bone-muscle crosstalk is rapidly evolving, our robust specimen storage will serve as a valuable resource for future work in this area. Ultimately, these data may also inform lean-mass research endeavors in related fields, such as exercise enhancement, sarcopenia prevention, and spaceflight safety.

Clinical recommendations to optimize bone outcomes following bariatric surgery include adequate consumption of calcium (1200–1500 mg/day), vitamin D (2000–3000 IU/day), and protein (60–75 g/day), along with regular weight-bearing exercise. [7,22] Increasingly, studies are being designed and implemented to test osteoprotective strategies, with many active trials registered on clinicaltrials.gov. Pertinent among these, are two exploring lifestyle-based therapies (NCT04193397, NCT04777305) studying either resistance training alone, or comparison of resistance training, aerobic training, or a combination of the two, and three (not including the STRONG BONES trial) are exploring pharmacotherapies (NCT04279392, NCT04087096, NCT04742010), using zoledronic acid or comparing zoledronic acid with denosumab. Two trials have been completed looking at mega-dosing vitamin D (NCT02092376) and the effects of tele-counseling behavioral counseling and a 12-week supervised exercise program (NCT03214471). As skeletal effects of bariatric surgery are multifactorial, future work in this area will likely aim to couple therapeutic strategies.

### 3.1 Strengths and limitations

Strengths of the STRONG BONES trial include the randomized controlled trial design and adequate power to examine treatment effects on the primary outcome, total hip aBMD. Pilot data informing active treatment selection (risedronate) and operational study flow are also strengths, as well as provision of robust biorepository specimen storage. However, limitations to the study design exist. Although DXA is the current clinical standard for diagnosis of osteoporosis ― with recent data supporting total hip aBMD as a surrogate outcome for fractures in future trials23 ― aBMD does not fully explain fracture risk prediction [65]. QCT provides additional bone quality information; however, both DXA and QCT have image resolution limitations. Ancillary work is planned to add high resolution peripheral quantitative computed tomography (HR-pQCT) scans to the study's bioimaging battery, providing state-of-the-art information on bone morphometry (e.g., trabecular number/spacing, cortical porosity) and remodeling. Finally, as free-living patients will be enrolled in this trial and bone turnover is influenced by multiple factors (including, but not limited to mechanical loading/unloading, hormonal perturbations, and nutritional intake), we cannot control for all potential confounders. That said, block randomization will be used to collect highly influential covariates.

## 4 Conclusion

While the benefits of bariatric surgery on weight and cardiometabolic health are significant and well-described, consideration of negative effects on lean mass including bone loss is important, particularly among older surgical patients. Results from this study will provide insight into the efficacy of risedronate use for attenuation of musculoskeletal tissue loss in this patient population. Along with the potential to influence clinical practice, mechanistic data exploring the counteractant effects of bisphosphonate therapy on SG-induced musculoskeletal loss will contribute to the growing field of bone-muscle crosstalk.

## Declaration of competing interest

The authors declare that they have no known competing financial interests or personal relationships that could have appeared to influence the work reported in this paper.
